# Eye movements as predictor of cognitive improvement after cognitive remediation therapy in patients with schizophrenia

**DOI:** 10.3389/fpsyt.2024.1395198

**Published:** 2024-04-16

**Authors:** Jiahui Zhu, Jinhao Li, Li Zhou, Lingzi Xu, Chengcheng Pu, Bingjie Huang, Qi Zhou, Yunhan Lin, Yajing Tang, Liu Yang, Chuan Shi

**Affiliations:** ^1^ Peking University Sixth Hospital, Peking University Institute of Mental Health, Beijing, China; ^2^ National Clinical Research Center for Mental Disorders, Peking University Sixth Hospital, Beijing, China; ^3^ NHC Key Laboratory of Mental Health, Peking University, Beijing, China; ^4^ Tianjin Anding Hospital, Mental Health Center of Tianjin Medical University, Tianjin, China; ^5^ Faculty of Education, East China Normal University, Shanghai, China; ^6^ Research and Development Department, Infinite Brain Technologies, Beijing, China; ^7^ The Affiliated Brain Hospital of Nanjing Medical University, Nanjing, China

**Keywords:** schizophrenia, cognitive intervention, cognitive improvement, prosaccades, antisaccades, free-viewing

## Abstract

**Aim:**

Baseline cognitive functions of patients predicted the efficacy of cognitive remediation therapy (CRT), but results are mixed. Eye movement is a more objective and advanced assessment of cognitive functions than neuropsychological testing. We aimed to investigate the applicability of eye movements in predicting cognitive improvement after patients with schizophrenia were treated with CRT.

**Methods:**

We recruited 79 patients with schizophrenia to complete 8 weeks of CRT and assessed their cognitive improvement outcomes. Eye movements were assessed by prosaccades, antisaccades, and free-viewing tasks at baseline, and neuropsychological tests in four cognitive domains were assessed before and after treatment to calculate treatment outcomes. Predictors of demographic information, clinical characteristics, and eye movement measures at baseline on cognitive improvement outcomes were analyzed using logistic regression analysis. We further compared the predictive performance between eye movement measurements and neuropsychological test regarding the effect of CRT on cognitive improvement, and explored factors that could be affect the treatment outcomes in different cognitive domains.

**Results:**

As operationally defined, 33 patients showed improved in cognition (improved group) and 46 patients did not (non-improved group) after CRT. Patients with schizophrenia being employed, lower directional error rate in antisaccade task, and lower the gap effect (i.e., the difference in saccadic latency between the gap condition and overlap condition) in prosaccade task at baseline predicted cognitive improvement in CRT. However, performance in the free-viewing task not associated with cognitive improvement in patients in CRT. Our results show that eye-movement prediction model predicted the effect of CRT on cognitive improvement in patients with schizophrenia better than neuropsychological prediction model in CRT. In addition, baseline eye-movements, cognitive reserve, antipsychotic medication dose, anticholinergic cognitive burden change, and number of training sessions were associated with improvements in four cognitive domains.

**Conclusion:**

Eye movements as a non-invasiveness, objective, and sensitive method of evaluating cognitive function, and combined saccadic measurements in pro- and anti-saccades tasks could be more beneficial than free-viewing task in predicting the effect of CRT on cognitive improvement in patients with schizophrenia.

## 1 Introduction

Cognitive impairment is one of the core features of schizophrenia (1) and occurs even before the onset of psychiatric symptoms (2). Patients with schizophrenia have widespread and prominent impairments in several major cognitive domains, and they perform 1–2 standard deviations lower than normal (3). Cognitive decline in schizophrenia has been shown to have an influential role in the functional outcome of patients (4). The anticholinergic burden of psychotropic medications is substantial, widespread, and adversely affects all cognitive domains (5), and there is insufficient evidence to support that medications significantly improve cognitive functioning in schizophrenia (6, 7). Therefore, it is crucial to find more effective techniques or methods to improve cognitive functioning in schizophrenia.

Cognitive remediation therapy (CRT) is behavioral training with the aim of sustained improvement of cognitive functioning that generalizes to social functioning (8). Over the last 20 years, it has been shown that CRT has a significant positive effect on schizophrenia, with small to moderate improvements in cognitive and social functioning (9, 10). However, not all schizophrenia patients benefit from CRT, as the effectiveness of the treatment largely depends on the characteristics of the individual and the treatment approach (11). Consequently, it is essential for clinicians to explore potential predictors of treatment response to CRT to identify individuals who are most likely to improve with treatment.

Several studies have explored the relationship between CRT response and sociodemographic and clinical characteristics in schizophrenia, but with mixed results. For example, some studies have shown that age is not associated with treatment outcomes (8, 12), whereas other, recent studies have proven that younger patients are more likely to benefit from CRT (13, 14). In terms of cognitive ability (such as memory, attention, and executive function) at baseline, some studies have shown that patients with lower cognitive ability at baseline experience greater benefit from CRT (15, 16), while other studies have reported the opposite results, that is, that higher baseline cognitive levels are associated with greater improvement in cognitive functioning (13, 17). In terms of neurobiological predictors, CRT response is related to cognitive reserve, and patients with greater layer thickness in the frontal and temporal lobes at baseline better respond to treatment (18). However, according to another study, cognitive reserve has a limited effect on treatment response (19). Hence, there is still a lack of sensitive and stable predictors of the outcomes of CRT.

Visual movement processing and visual perception impairment are still some of the most significant abnormalities in schizophrenia (20). The ability of saccades and visual scanning is usually measured by pro- or antisaccades and free-viewing tasks, respectively. The abnormal eye movements in schizophrenia in free-viewing and pro- or antisaccades have been recognized for a long time (21, 22), and they are considered one of the most promising biomarkers for schizophrenia. Neuroimaging studies have also shown that visual behavioral deficits and schizophrenia share several involved regions of the brain, including but not limited to, prefrontal cortex, visual cortex, parietal cortex, anterior cingulate cortex, striatum, superior colliculus, and supplementary eye field (23, 24). Eye movement assessment is noninvasive, and provides an insightful “window” with an indirect way to measure the functioning of our brains.

Eye movement as a neurophysiological indicator, and associated with higher cognitive processes and psychomotor functions (25, 26). Prosaccades are recognized as reflexive saccades, where participants need bottom-up attention to complete this paradigm (27), while antisaccades involve inhibition and cognitive control (28, 29). These results strongly suggest a relationship between abnormal eye movements and cognitive deficits in schizophrenia. It has been shown that the directional error rate and antisaccade latency in patients with schizophrenia during the antisaccade tasks are related to performance on the Digit Sequencing and Symbol Coding Test, respectively (30). Higher cognitive processes involved sensorimotor and visual information switching abilities have been demonstrated in the antisaccade task (31). Patients with schizophrenia in symbolic coding tasks with poor visual search strategies, they made more visits to key areas as well as spending more time searching for target symbols (32). Additionally, traditional neuropsychological measures of change after cognitive interventions shown a pattern of modest improvement (33, 34), but eye movement indicators demonstrated a mixed pattern of beneficial and adverse treatment-related effects (35, 36). In particular, eye movement indicators are more sensitive compared to neuropsychological test for the diagnose cognitive impairments (37).

Despite the fact that there are some studies that have explored the clinical characteristics and cognitive function-related predictors of CRT in schizophrenia, the results and factors remain inconsistent and incomplete. Moreover, previous studies have not included neurophysiological biomarkers that correlate with cognition, are more sensitive, and may be sufficient to more accurately identify candidates for CRT. In our study, we recruited schizophrenia patients with cognitive impairment to investigate the predictors of the improvement of cognitive functioning in patients after CRT. We compared the predictive performance between eye movement measures and neuropsychological test. We further explored the demographic information, clinical characteristics, and eye movements associated with cognitive treatment outcome and improvement in different cognitive domains. We hypothesized that, in addition to previous factors, baseline eye movement characteristics among patients may be a potential biomarker of CRT treatment outcome.

## 2 Methods

**Figure 1:**
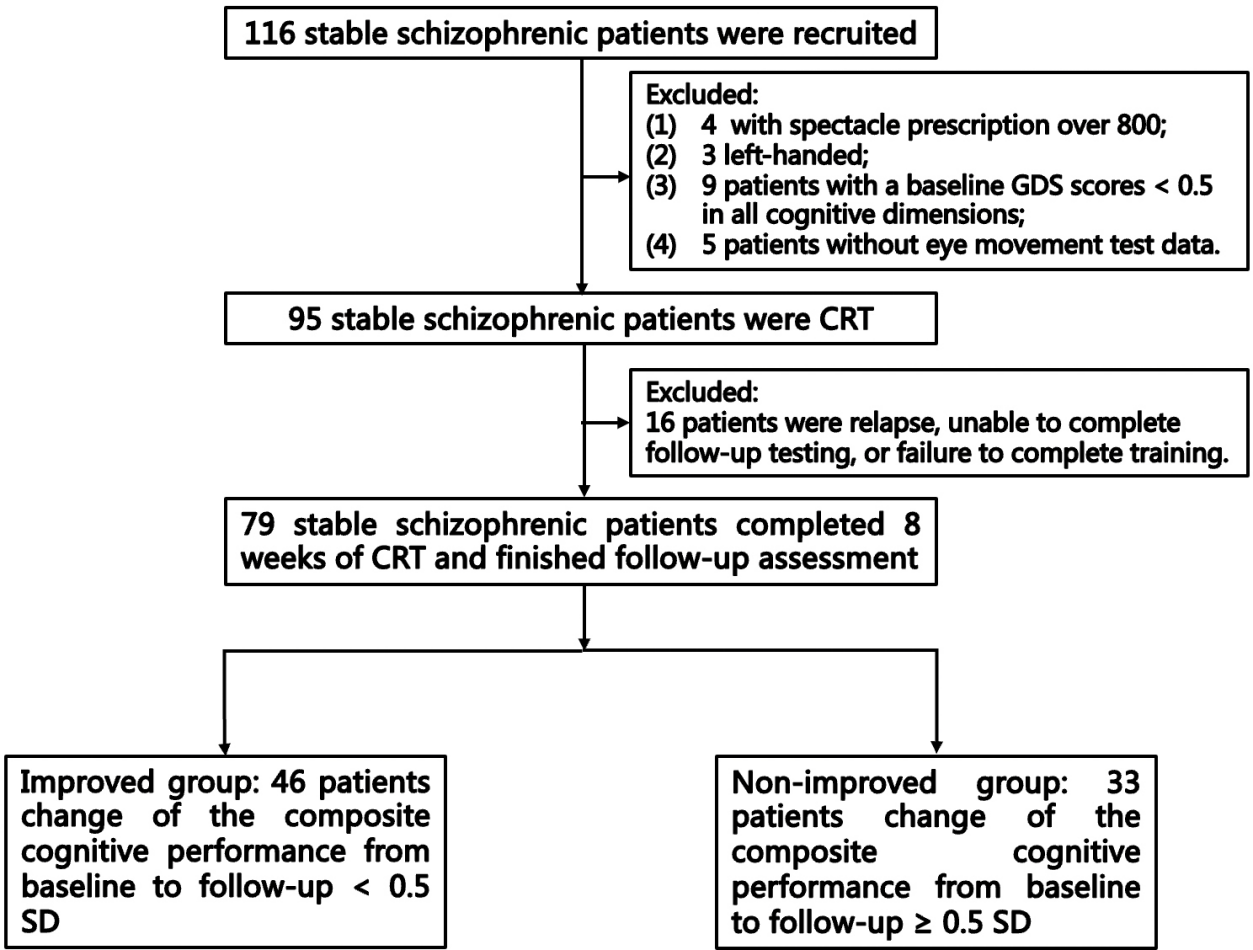
Flow diagram of selection and follow-up of participants.

This single-arm pre–post study was registered in the Chinese Clinical Trial Registry (ChiCTR-2200062704). All of the participants diagnosed with schizophrenia who consented and fulfilled the inclusion criteria completed the 8-week CRT. The qualified examiners were trained to obtain data. Participants completed assessments of psychiatric symptoms, cognitive functioning, and eye movement behavior at baseline, and completed assessments of psychiatric symptoms and cognitive functioning at follow up. The flow diagram of the selection and follow-up of participants is shown in **Figure 1**. This study focused on the cognition outcomes, and other outcomes will be reported in a future paper.

### 2.1 Participants

The participants were recruited from Peking University Sixth Hospital and assessed by outpatient physicians. The inclusion criteria for the patients with schizophrenia were as follows: (1) diagnosis of schizophrenia confirmed by two psychiatrists (38); (2) age ≥ 18 and ≤ 40 years; (3) ≥ 5 years of education; (4) the dose and type of antipsychotics remaining stable for at least 1 month prior to the evaluation, and the severity of several items in the Positive and Negative Syndrome Scale (PANSS) (39) being less than 5 points (including P1 (Delusions), P2 (Conceptual disorganization), P3 (Hallucinatory behavior), P5 (Grandiosity), P6 (Suspiciousness/persecution), and G9 (Unusual thought content)); (5) right-handedness; (6) the Global Deficit Score (GDS) at baseline for at least one cognitive domain ≥ 0.5. Exclusion criteria were as follows: (1) myopia degree of more than 800 degrees, or clinical history of ophthalmic diseases; (2) history of substance abuse; (3) intellectual disability, organic cerebral diseases, or other severe organic disorders; (4) plan to change medication during the intervention; (5) any physical interventions within the past 6 months. Except for four patients, all of the patients were treated with antipsychotic medications before the intervention and maintained until follow-up. The dosage and type of antipsychotic drugs could be modified depending on the patients’ symptoms. Benzodiazepines were allowed according to the psychiatrists’ judgment.

The study was approved by the Ethics Committee of the Peking University Sixth Hospital (Approval No. 2022-38, 5^th^ of July 2022). All of the participants signed the informed consent form prior to the assessment.

### 2.2 Assessment

#### 2.2.1 Clinical assessment

The PANSS (39) and the Global Assessment Function (GAF) (40) scale were used to assess the patients’ symptom severity and psychosocial function, respectively, at baseline and follow-up. The dose of antipsychotics used during the intervention was calculated as chlorpromazine equivalent (CPZ-eq) (41). A modified version of the Anticholinergic Cognitive Burden (ACB) scale was used to calculate the total anticholinergic cognitive burden in drug treatment regimen of each patient (5). The baseline values were subtracted from the follow-up values to calculate the change in CPZ-eq and ACB scores of the participants after the intervention.

#### 2.2.2 Cognitive assessment

The four cognitive domains were assessed as follows: (1) Speed of processing: Trail Making Test-A (TMT-A), Symbol Coding Test (SC), and Stroop Color and Word tests (SCT; SWT); (2) Working memory: Digit Span Test (DS) and Spatial Span Test (SST, WMS-III); (3) Attention: Continuous Performance Test (CPT); and (4) Executive function: Mazes Test (NAB-MAZES) and Stroop Color-Word Test (SCWT). The TMT-A, SC, DS, and CPT were assessed using the Chinese Brief Cognitive Test (C-BCT), which exhibited good internal consistency and test–retest reliability, making it an effective assessment tool for schizophrenia (42).

To correct for demographic factors affecting cognitive performance, the raw score was transformed into a T-score in each test (Mean: 50; SD: 10) adjusted by Chinese norms (42, 43). The higher the T-score, the better the participants completed the test. The T-score was transformed into GDS, and the correspondence between the T-score and GDS was as follows: T-scores ≥ 40, 39–35, 34–30, 29–25, 24–20, and ≤ 19 equaled GDS of normal = 0, mild = 1, mild to moderate = 2, moderate = 3, moderate to severe = 4, and severe = 5, respectively (44). Then, the T-score and GDS of each cognitive domain were calculated by averaging all of the tests in that specific cognitive domain. Finally, the T-score and GDS of the composite cognition were calculated by averaging the scores of the four cognitive domains. A GDS score of 0.5 or higher for each cognitive domain was defined as a deficit in that cognitive domain. All of the patients who participated in the CRT had at least one deficit cognitive domain before intervention.

In this study, cognitive improvement was operationally defined using criteria from previous studies. The intervention was considered to have had a meaningful effect only if the composite cognitive performance change of patients from baseline to follow-up was ≥ 0.5 SD, and this subset of patients was defined as the improved group (45). In contrast, patients with the composite cognitive score improvement of < 0.5 SD were defined as the non-improved group (46).

#### 2.2.3 Eye movement recording and processing

The eye movements were recorded using the Eyelink 1000 Plus system (SR Research, Ontario, Canada, 1000 Hz). During the experiment, the eye-movement tasks were displayed on a 19-inch monitor (1024 × 768 resolution; 60 Hz), and the distance between the participant’s eyes and the screen was 70 cm. At each visit, each participant completed prosaccade, antisaccade, and free-viewing tasks in order, and the operator gave a description of each task before starting. Each block was calibrated using a 5-point target display before starting, and then verified on the same display. Each trial started with a central fixation (1-degree diameter) on a black background, duration ranging from 500 to 1000 ms (pseudo-randomized sequence). In the pro- and antisaccade tasks, stimulus point (white square shapes, 1 degree × 1 degree) presented on a black background for 1000 ms.

**Figure 2:**
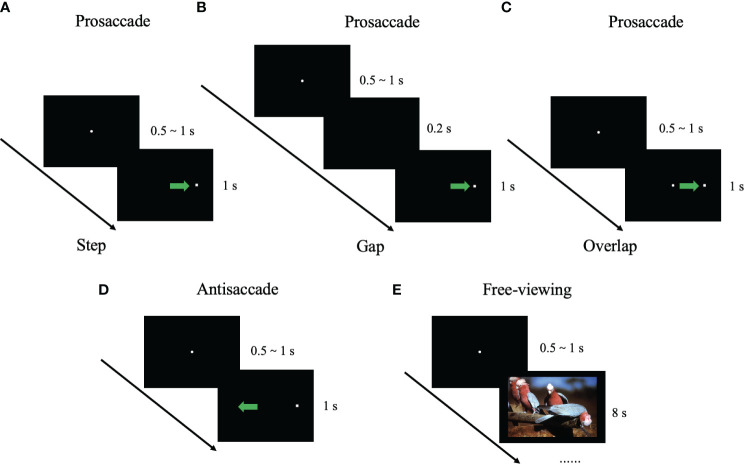
Illustration of eye-movement tasks. The filled circle represents the center fixation point. The white square shape represents the stimulus point. The blue arrow represents the required saccade trajectory. **(A)** Prosaccade task (step condition); **(B)** Prosaccade task (gap condition); **(C)** Prosaccade task (overlap condition); **(D)** Antisaccade task; and **(E)** Free-viewing task.

The prosaccade task consisted of three blocks of 48 trials. Before the task started, the participants were told to produce a visual scan of the target point as fast as possible. After the central fixation has disappeared, the target appeared at 8° from the center fixation, and the central fixation point disappeared before the target appearance 200 ms (Gap), after the target appearance (Step), or simultaneously with the target (Overlap). (**Figures 2** A. Step, B. Gap, C. Overlap) The following eye movement measures were obtained: (1) Mean latency of the first prosaccade in each trial (overlap/gap/step); (2) Gap effect, the difference in saccadic latency between the gap condition and overlap condition in the prosaccade task; (3) Mean peak velocity of the first prosaccade in each trial; and (4) Mean primary saccade gain in the prosaccade task, determined as the first saccadic amplitude divided by the distance between the target and the center.

The antisaccade task consisted of three blocks of 48 trials. Before the task started, the participants were told to look in the opposite direction of the target point rather than at the target itself. After the central fixation has disappeared, the target appeared 8° from the center fixation with step condition. (**Figure 2D**) The following eye movement measures were obtained: (1) Mean correct latency, i.e., the reaction time to the onset of the first correct antisaccade; (2) Mean error rate, i.e., the percentage of wrong-direction saccade produced; (3) Mean correction rate, i.e., the percentage of saccade to the mirrored position toward the target point after wrong-direction antisaccade.

The free-viewing task consisted of one block of 35 trials. Each image was presented for 8 s, and the participants were free to view the images. All images were designed to appeared in a pseudo-randomized sequence and are presented with the same image for participants. The types of images used involved natural environments, everyday objects, buildings, food, and animals (7 images for each type, for a total of 35 images). All of the images were selected from the International Affective Pictures System (47). (**Figure 2E**) For each image, we measured the scanpath length, the number of the fixations and saccades, the mean duration and mean peak velocity of the saccades, the median fixation duration and saccade amplitude. Finally, we calculated the average of the above measures for each participant.

Raw eye-movement position data were segmented into the saccades and blinks using Eyelink Data Viewer (version 4.2.1). In this study, we only included macrosaccades for calculation, and the threshold for identification was 1° (48). Then, we eliminated all of the start or stop positions off-screen as well as all saccades labeled as blinks. Finally, all of the data were transferred to another PC for offline analysis using the tidyverse software package (version 1.3.1) in R 4.1.0.

### 2.3 Training

Training was conducted on computerized cognitive training software (IBT-SC01) developed by Infinite Brain Technology, Inc. The main design elements, the user interface, and the training game tasks interface of the software are presented in the **Supplementary Material**. The software can be used independently by patients, not limited to place, and is an automated system that provides voice guidance and practice for patients to understand the rules of the task. The IBT-SC01 consists of 13 gaming tasks across six cognitive domains (speed of reaction, attention, memory, executive function, logical reasoning, and thinking ability). The IBT-SC01 course of 56 sessions (30–45 minutes per session) was scheduled so that each patient completed one session per day for a total of 8 weeks of training. The system automatically assigned daily training tasks, and the patients were required to complete four game tasks in different cognitive domains. The difficulty of the games was adaptive, and the system matched the difficulty of training in the next session to the patient’s current cognitive ability. A trained professional was responsible for the daily training progress of the patients.

### 2.4 Statistical analysis

We used SPSS 21.0 (IBM Corp., Armonk, NY, USA) for statistical analysis of the data. In the general descriptive analysis, Shapiro–Wilk (*S–W*) test was used for continuous variables. If data conformed to normal distribution, they were represented as mean (standard deviation); otherwise, they were represented as median (interquartile range). Categorical variables were expressed as frequency (percentage). Comparison of demographic information, clinical characteristics, cognitive performance, and eye-movement measures between the cognitive improved and non-improved groups was based on independent-samples *t* test (or Mann–Whitney *U* test) and chi-square test (or Fisher’s exact test). To analyze the changes in clinical characteristics and cognitive domains’ T-scores after CRT, paired-sample *t* test was used for normally distributed variables, and Wilcoxon test was used for non-normally distributed variables. To quantify the magnitude of change after treatment, effect sizes for each cognitive domain and clinical symptom were assessed using Cohen’s *d.*

Spearman correlation analysis was used to investigate the correlation between baseline cognitive T-scores and T-scores changes (ΔT-score), and baseline eye-movement characteristics (corrected by the Bonferroni correction). The possible predictors of CRT improvement outcomes were identified by binary logistic regression analysis, and the associations between these factors and outcomes were described as odds ratios (OR) and 95% confidence intervals (95% CI). All variables included in the binary logistic regression analysis that were significantly different in the comparison between the two groups or were associated with cognitive T-scores. In the first model, we included demographic, baseline clinical characteristics, neuropsychological test, and eye-movement measures. In order to compare the predictive performance of eye movements and neuropsychological test, other two regression models were performed. In the second model, we only included baseline cognitive T-scores in neuropsychological test. In the third model, we only included baseline eye-movement measures. We use the receiver–operator curve (ROC) analysis by MedCalc statistical software v20 (MedCalc Software Ltd, Ostend, Belgium) to evaluate the predictive performance of prediction probability between eye-movements prediction model (second model) and neuropsychological test prediction model (third model). Linear regression analysis was used to explore factors that may influence for specific cognitive domains, including variables that were entered into the logistic regression model and factors were demonstrated in previous studies to influence CRT outcome.

Considering that their number could not exceed 1/10 of the total sample, it was specified that only variables with *p* < 0.05 in the comparison between the improved and non-improved groups could enter the regression model.

## 3 Results

A total of 95 participants were treated with CRT based on the inclusion and exclusion criteria. Sixteen participants dropped out of the study because they suffered a relapse that made them unable to complete the training or they did not complete the follow-up interviews. Finally, a total of 79 patients completed the 8 weeks of CRT. The improved group included 46 patients, the non-improved group included 33 patients, and 25 patients showed cognitive decline. At baseline, three patients had not taken any psychiatric medications for at least 1 month. There were seven patients who did not complete the free-viewing task, and one patient did not complete the antisaccade task at baseline. Except for one patient who completed only 23 sessions, all of the other patients completed at least 60% of the sessions.

### 3.1 Intergroup comparison of demographic and clinical characteristics, cognitive T-score and eye-movement measures at baseline

Baseline demographic and clinical characteristics of the non-improved and improved groups are demonstrated in **Table 1**. There were no significant differences in age, sex, education, marital status, and drinking between the two groups. The percentage of employed in the improved group was higher (χ2 = 12.907 *p* < 0.001) than that in the non-improved group. Other clinical characteristics of the patients are also listed in **Table 1**.

**Table 1:** Baseline demographic and clinical characteristics of patients in the improved group and the non-improved group.

Variable	Non-improved group (N = 46)	Improved group (N = 33)	*χ2/t/Z*	*p*
	Mean ± SD/n (%)/Median (IQR)	Mean ± SD/n (%)/Median (IQR)		
Age (years)	27.85 ± 6.69	27.15 ± 4.89	0.534	0.595
Sex			0.583^a^	0.445
Male	13 (28.3)	12 (36.4)		
Female	33 (71.7)	21 (63.6)		
Education (years)	14.84 ± 2.59	15.39 ± 2.14	−1.012	0.315
Employment status			12.907^a^	<0.001^***^
Employed	12 (26.1)	22 (66.7)		
Unemployed	34 (73.9)	11 (33.3)		
Marital status			0.007^a^	0.934
Married	3 (6.5)	2 (6.1)		
Unmarried	43 (93.5)	31 (93.9)		
Drinking			0.149^a^	0.700
Non-/ex-	44 (95.7)	30 (90.9)		
Current	2 (4.3)	3 (9.1)		
Duration of illness (months)	61.74 ± 53.45	73.55 ± 60.07	−0.919	0.361
DUP (months)	6.33 ± 10.93	10.28 ± 20.18	−1.121	0.266
Hospitalization (n)	1.43 ± 1.44	1.30 ± 1.47	0.398	0.692
Recurrence (n)	1.26 ± 1.50	0.97 ± 0.98	0.975	0.332
Family history of mental disorders	7 (0.2)	1 (0.03)	1.940	0.164
Positive PANSS score	10.76 ± 3.88	9.18 ± 3.21	1.916	0.059
Negative PANSS score	12.96 ± 4.72	11.94 ± 4.69	0.947	0.347
General psychopathological PANSS score	23.26 ± 4.80	21.36 ± 4.98	1.706	0.092
Total PANSS score	46.89 ± 10.31	42.52 ± 9.98	1.886	0.063
GAF score	70.91 ± 10.59	75.42 ± 9.80	−1.925	0.058
CPZ equivalent dose (mg/day)	518.86 (300.23, 859.92)	275.23 (150.00, 424.40)	−3.108^b^	0.002^**^
ACB score	4.00 (3.00, 5.00)	3.00 (1.00, 4.00)	−2.523^b^	0.012^*^
Number of training sessions (n)	46.39 ± 12.16	49.76 ± 8.03	−1.387	0.169

^*^ p < 0.05, ^**^p < 0.01, ^***^p < 0.001;

DUP, Duration of untreated psychosis; PANSS, Positive and Negative Symptom Scale; GAF, Global Assessment of Functioning score; CPZ, Chlorpromazine; ACB, Anticholinergic Cognitive Burden scale; ^a^ Fisher’s exact test; ^b^ Mann–Whitney U test.

Baseline T-score of each cognitive domain, cognitive composite scores, composite GDS, and eye-movement measures of the patients are presented in **Table 2**. At baseline, only the executive function and cognitive composite T-score were significantly different between the two groups (*p =* 0.003, *p* = 0.047, respectively). Compared with the non-improved group, the patients in the improved group had lower the gap effect, higher peak velocities, and higher primary saccade gain during prosaccade task (*p =* 0.031, *p* = 0.008, *p* = 0.036, respectively). Compared with the non-improved group, the patients in the improved group had lower error rate and higher correction rate during antisaccade task (*p* = 0.001, *p* = 0.003, respectively). There were no significant intergroup differences in other eye-movement measures.

**Table 2:** Baseline cognitive performance and eye-movement measures of the cognitive improved and non-improved groups.

	Non-improved group (N = 46)	Improved group (N = 33)	*t/Z*	*p*
	Mean ± SD/Median (IQR)	Mean ± SD/Median (IQR)		
Baseline T-score				
Processing speed	42.78 ± 6.50	45.43 ± 5.81	−1.866	0.066
Attention	47.72 ± 5.94	48.76 ± 5.57	−0.787	0.434
Working memory	43.49 ± 6.75	43.26 ± 5.34	0.162	0.871
Executive function	37.25 ± 6.28	41.83 ± 7.08	−3.033	0.003^**^
Cognitive composite scores	42.81 ± 4.36	44.82 ± 4.42	−2.014	0.047^*^
Global deficit score	0.65 ± 0.52	0.47 ± 0.38	1.649	0.103
Prosaccade task				
Latency (gap) (ms)	151.35 ± 24.65	154.65 ± 29.68	−0.539	0.591
Latency (overlap) (ms)	188.82 ± 35.07	182.07 ± 39.04	0.805	0.423
Latency (step) (ms)	167.67 ± 26.44	168.53 ± 33.22	−0.128	0.899
Gap effect (ms)	37.47 ± 20.87	27.41 ± 18.91	2.196	0.031^*^
Peak velocity (°/s)	304.75 ± 52.24	340.75 ± 64.70	−2.733	0.008^**^
Primary saccade gain	0.79 ± 0.08	0.82 ± 0.07	−2.139	0.036^*^
Antisaccade task				
Correct latency (ms)	338.32 ± 71.99	314.64 ± 59.85	1.529	0.131
Error rate	0.44 ± 0.19	0.28 ± 0.22	3.335	0.001^**^
Correction rate	0.18 (0.16, 0.26)	0.28 (0.21, 0.39)	-2.977^a^	0.003^**^
Free-viewing task				
Fixation number (n)	19.45 ± 4.55	19.42 ± 3.99	0.036	0.971
Fixation duration (ms)	304.16 (271.18, 363.62)	289.16 (260.07, 362.71)	−0.871^a^	0.384
Saccade number (n)	15.62 ± 4.93	15.69 ± 4.09	−0.064	0.949
Saccade duration (ms)	33.27 ± 5.07	35.50 ± 5.12	−1.804	0.076
Saccade peak velocity (°/s)	183.93 ± 43.77	192.72 ± 35.33	−0.889	0.377
Saccade amplitude (°)	3.16 (2.63, 3.75)	3.24 (2.86, 3.68)	−0.294 ^a^	0.769
Scanpath length	110.26 ± 31.08	109.68 ± 18.42	0.098	0.922

^*^ p < 0.05, ^**^p < 0.01;

^a^ Mann–Whitney U test.

### 3.2 Correlation analysis between baseline cognitive T-scores and ΔT-score, and baseline eye-movement measures in schizophrenia patients

**Figure 3:**
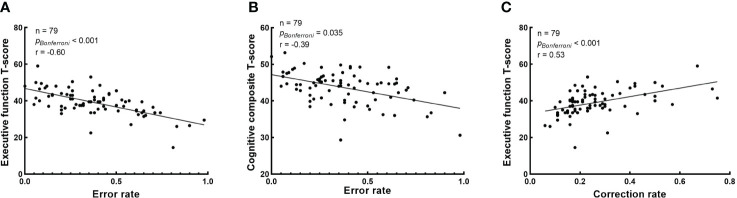
Correlation analysis between baseline cognitive T-scores and baseline eye-movement measures. The Bonferroni corrected p-values and r in the correlation analysis are presented, and raw p-values were multiplied by 80 for correlation. **(A)** The correlation between the error rate in antisaccade task and executive function T-score; **(B)** The correlation between the error rate in antisaccade task and cognitive composite T-score; and **(C)** The correlation between the correction rate in antisaccade task and executive function T-score.

The correlation between baseline cognitive T-scores and baseline eye-movement measures in schizophrenia patients is shown in **Figure 3**. After Bonferroni correction, correlational analysis demonstrated that baseline error rate in antisaccade negatively correlated with executive function and cognitive composite T-score (r = -0.60, *p* < 0.001 and r = -0.39, *p* = 0.035, respectively) (**Figures 3A, B**); Baseline correction rate in antisaccade positively correlated with executive function T-score (r = 0.53, *p* < 0.001) (**Figure 3C**). However, we didn’t find the correlation between baseline eye-movement measures in the free-viewing task and cognitive functioning in patients.

**Figure 4:**
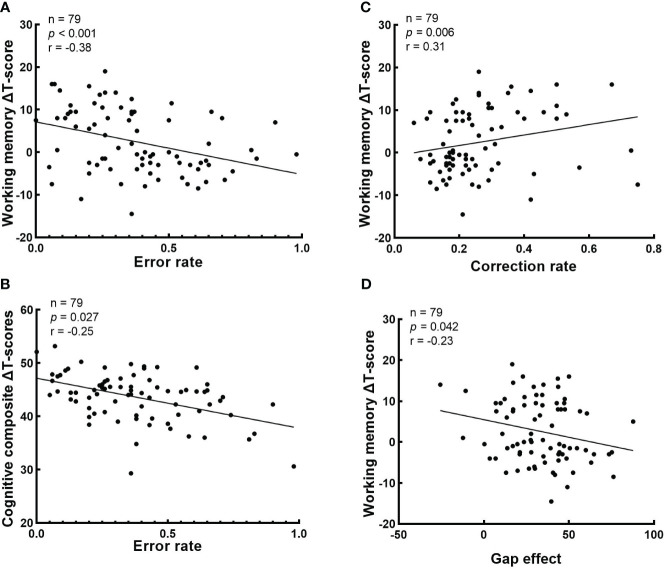
Correlations analysis between cognitive ΔT-score and baseline eye-movement measurements. The values of p, r in the correlation analysis is presented. **(A)** The correlation between the error rate in antisaccade task and working memory ΔT-score; **(B)** The correlation between the error rate in antisaccade task and cognitive composite ΔT-score; **(C)** The correlation between the correction rate in antisaccade task and working memory ΔT-score; and **(D)** The correlation between the gap effect in prosaccade task and working memory ΔT-score. .

The correlations between cognitive ΔT-score and baseline eye-movement measurements in schizophrenia patients is shown in **Figure 4**. The correlation analysis shown that baseline error rate in antisaccade negatively correlated with working memory ΔT-score and cognitive composite ΔT-score (r = -0.38, raw *p* < 0.001 and r = -0.25, raw *p* = 0.027, respectively) (**Figures 4A, B**); Baseline correction rate in antisaccade positively correlated with working memory ΔT-score (r = 0.31, raw *p* = 0.006) (**Figure 4C**). Baseline the gap effect in prosaccade negatively correlated with working memory ΔT-score (r = -0.23, raw *p* = 0.042) (**Figure 4D**). However, any correlation results did not reach a significant level after Bonferroni correction.

### 3.3 Comparison of clinical characteristics and cognitive domains’ T-scores of schizophrenia patients pre- and post-CRT

The comparison of clinical characteristics and cognitive performance in the schizophrenia patients before and after 8 weeks of CRT is shown in **Table 3**. There were no significant changes in PANSS scores, GAF scores, CPZ-eq, and ACB scores after CRT in the schizophrenia patients. In addition, compared with baseline, we found improvements in processing speed (*d*, 0.29, *p* < 0.001), attention (*d*, 0.26, *p =* 0.008), working memory (*d*, 0.36, *p =* 0.003), executive functioning (*d*, 0.33, *p* < 0.001), and overall cognitive (*d*, 0.43, *p* < 0.001) T-score after 8 weeks of intervention.

**Table 3:** Clinical characteristics and cognitive domains’ T-scores of patients with schizophrenia pre- and post-CRT.

	Baseline (N = 79)	Follow-up (N = 79)	*t^a^/Z*	*Cohen’s d*	*p*
	Mean ± SD/Median (IQR)	Mean ± SD/Median (IQR)			
Positive PANSS score	10.10 ± 3.68	9.91 ± 3.24	1.149	0.05	0.254
Negative PANSS score	12.53 ± 4.71	12.32 ± 4.36	1.087	0.05	0.280
General psychopathological PANSS score	22.47 ± 4.94	22.10 ± 4.90	1.703	0.08	0.093
Total PANSS score	45.06 ± 10.34	44.23 ± 9.65	1.975	0.08	0.052
GAF score	72.80 ± 10.45	73.16 ± 9.54	−1.063	0.04	0.291
CPZ equivalent dose (mg/day)	375.37 (200.00, 750.45)	305.39 (200.00, 749.80)	−1.784^b^		0.074
ACB score	4.00 (1.75, 5.00)	3.00 (1.00, 5.00)	−1.706^b^		0.088
Processing speed T-score	43.89 ± 6.32	45.66 ± 5.70	−6.681	0.29	<0.001^***^
Attention T-score	48.15 ± 5.78	49.49 ± 4.63	−2.707	0.26	0.008^**^
Working memory T-score	43.39 ± 6.16	46.00 ± 8.18	−3.035	0.36	0.003^**^
Executive function T-score	39.16 ± 6.96	41.95 ± 9.49	−3.879	0.33	<0.001^***^
Cognitive composite scores T-score	43.65 ± 4.47	45.78 ± 5.46	−5.589	0.43	<0.001^***^

^**^ p < 0.01, ^***^p < 0.001;

PANSS, Positive and Negative Symptom Scale; GAF, Global Assessment of Functioning score; CPZ, Chlorpromazine; ACB, Anticholinergic Cognitive Burden scale; ^a^ Paired t test; ^b^ Wilcoxon test.

### 3.4 Predictors of cognitive improvements after CRT in schizophrenia patients, and comparison between the neuropsychological test prediction model and eye-movements prediction model

Three different models of binary logistic regression were tested, and detailed results for each model are shown in **Table 4**. The results of the first model analysis showed that patients being employed (OR: 3.478; 95% CI: 1.096, 11.038, *p* = 0.034) was a favorable factor for cognitive improvement after CRT, while a higher baseline error rate in the antisaccade (OR: 0.018; 95% CI: 0.001, 0.362, *p* = 0.009) and the gap effect in the prosaccade task (OR: 0.964; 95% CI: 0.934, 0.994, *p* = 0.020) seemed to be unfavorable factors. The results of the second model analysis showed that a higher baseline executive function T-score (OR: 1.122; 95% CI: 1.033, 1.219, *p* = 0.006) was a favorable factor for cognitive improvement after CRT. The results of the third model analysis showed that a higher baseline peak velocity in prosaccade (OR: 1.011; 95% CI: 1.001, 1.021, *p* = 0.025) was a favorable factor, while a higher baseline error rate in the antisaccade (OR: 0.013; 95% CI: 0.001, 0.240, *p* = 0.003) and the gap effect in the prosaccade task (OR: 0.969; 95% CI: 0.940, 0.999, *p* = 0.040) to be the unfavorable factors for cognitive improvement after CRT.

**Table 4:** Predictors of cognitive improvement after CRT in patients with schizophrenia (backward logistic regression).

Variable	*B*	OR (95% CI)	*p*
Model 1: baseline demographic, clinical characteristics, neuropsychological test, and eye-movements			
Employment status	1.246	3.478 (1.096, 11.038)	0.034
Error rate ^a^	−3.991	0.018 (0.001, 0.362)	0.009
Gap effect ^b^	−0.037	0.964 (0.934, 0.994)	0.020
Model 2: baseline neuropsychological test			
Baseline executive function T-score	0.115	1.122 (1.033, 1.219)	0.006
Constant	-4.888		0.004
Model 3: baseline eye-movements tests			
Gap effect ^b^	-0.032	0.969 (0.940, 0.999)	0.040
Peak velocity ^b^	0.011	1.011 (1.001, 1.021)	0.025
Error rate ^a^	-4.318	0.013 (0.001, 0.240)	0.003

Model 1 input variables included: employment status, CPZ-eq, ACB score, baseline T-score of executive function and cognitive composite scores, gap effect, peak velocity, and primary saccade gain during prosaccade task, error rate and correction rate during antisaccade task; Model 2 input variables included: baseline T-score of executive function and cognitive composite scores; Model 3 input variables included: gap effect, peak velocity, and primary saccade gain during prosaccade task, error rate and correction rate during antisaccade task;

The Non-improved group and improved group are coded as 0 and 1, respectively. OR, odds ratios; CI, confidence intervals; ^a^ antisaccade task; ^b^ prosaccade task.

**Figure 5:**
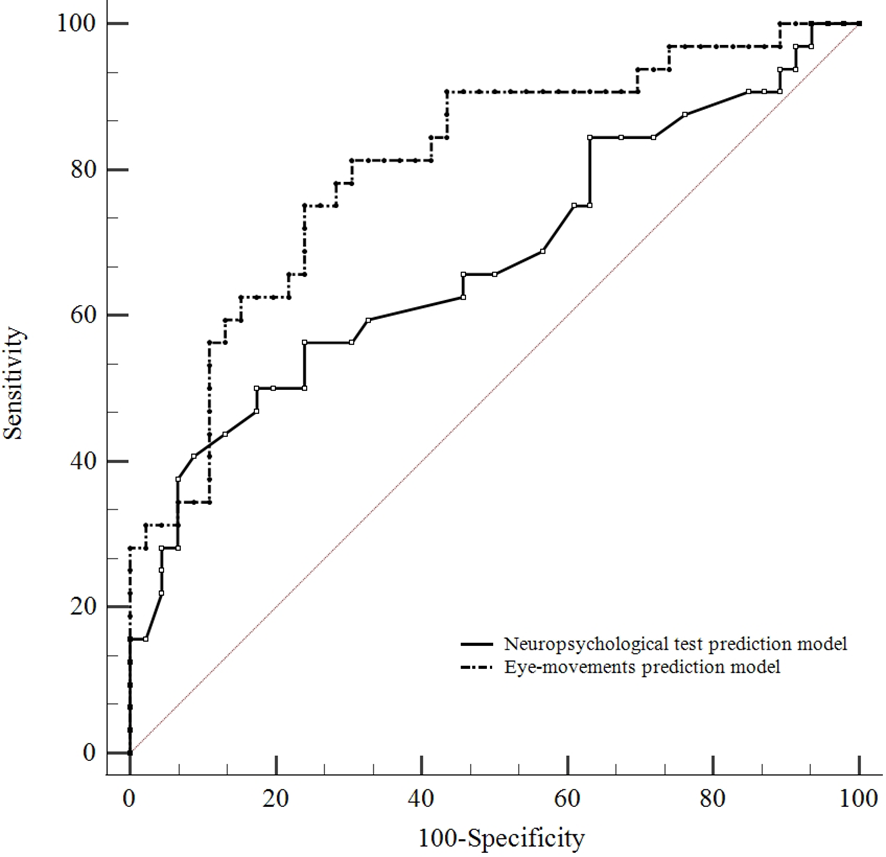
Comparison of predictive performance between the neuropsychological test and eye-movements prediction model. ROC curve analysis shows identification of CRT improved patients and CRT non-improved patients is better by using the eye-movements prediction model (AUC = 0.804, 95% CI 0.705 to 0.904) than the neuropsychological test model (AUC = 0.676, 95% CI 0.550 to 0.802). A pairwise comparison of ROC curves: eye-movements prediction model vs. neuropsychological test prediction model (p = 0.041).

We compared the predictive performance between the neuropsychological test prediction model and eye-movements prediction model by employing ROC curve analysis. The ROC curve analysis shows identification of CRT improved patients and CRT non-improved patients is better by using the eye-movements prediction model (AUC = 0.804, 95% CI 0.705 to 0.904, Youden index at the optimal cutoff point = 0.511, sensitivity = 75.00%, specificity = 76.10%) than the neuropsychological test prediction model (AUC = 0.676, 95% CI 0.550 to 0.802, Youden index at the optimal cutoff point = 0.326, sensitivity = 50.00%, specificity = 82.60%; A pairwise comparison of ROC curves by z test: eye-movements prediction model vs. neuropsychological test prediction model, *p* = 0.041). (**Figure 5**)

### 3.5 Predictors of cognitive improvements that influenced the specific cognitive domains after CRT in schizophrenia patients

The predictors that influenced the cognitive ΔT-score of specific cognitive domains are shown in **Table 5**. In the processing speed domain, a higher baseline T-score, error rate in the antisaccade, and the increase in ACB score were unfavorable factors for T-score improvement. In the attention domain, a higher baseline T-score was an unfavorable factor for T-score improvement. In the working memory domain, a higher baseline T-score, error rate in the antisaccade, CPZ-eq, and the gap effect in the prosaccade were unfavorable factors, and the greater number of training sessions was a favorable factor for T-score improvement. In the executive function domain, patients in employment and the greater number of training sessions were favorable factors, whereas the increase in ACB score was an unfavorable factor for T-score improvement.

**Table 5:** Linear regression analyses of the factors influencing changes in cognitive domains after CRT in patients with schizophrenia.

ΔT-score of cognitive domains	Factors	*B*	*SE*	*Beta*	*p*
Processing speed	T-score at baseline	−0.155	0.042	−0.416	<0.001^***^
	Error rate ^a^	−2.441	1.194	−0.222	0.045^*^
	ΔACB score	−0.674	0.245	−0.295	0.008^**^
Attention	T-score at baseline	−0.457	0.068	−0.614	<0.001^***^
Working memory	T-score at baseline	−0.429	0.112	−0.351	<0.001^***^
	Error rate ^a^	−12.643	3.136	−0.362	<0.001^***^
	CPZ equivalent dose (mg/day)	−0.007	0.002	−0.277	0.004^**^
	Number of training sessions	0.188	0.063	0.268	0.004^**^
	Gap effect ^b^	−0.091	0.033	−0.249	0.007^**^
Executive function	ΔACB score	−2.140	0.618	−0.350	0.001^**^
	Employment status	4.376	1.300	0.342	0.001^**^
	Number of training sessions	0.169	0.060	0.286	0.006**

^*^ p < 0.05, ^**^p < 0.01, ^***^p < 0.001;

CPZ, Chlorpromazine; ACB, Anticholinergic Cognitive Burden scale; ^a^ antisaccade task; ^b^ prosaccade task.

## 4 Discussion

We found that all four cognitive dimensions of patients with schizophrenia showed significant improvements after CRT relative to baseline. Here, we used a relatively conservative definition of cognitive improvement (≥ 0.5 SD) because it was sufficiently perceptible to patients and physicians to demonstrate that the change did not occur by chance (49). For the first time, we considered eye movement in investigating predictors of CRT. We found that baseline eye-movement characteristics in schizophrenia patients predicted cognitive improvement after CRT.

After the CRT, there were 46 (58%) patients who improved in at least one cognitive domain, but none of the patients had improvements in all cognitive domains. This result is a little better than that in previous studies (46.2% and 49.5%) (49, 50). These differences in treatment outcomes may derive from the different cognitive domains included between studies, and if more cognitive assessments were included our percentage of improvement could change. According to the results of a meta-analysis and review, no matter which computerized cognitive training system or country, a small to moderate effect of cognitive therapy on the primary outcome is observed (10, 51). Our study showed similar results (*d*, 0.29–0.43). We also found that CRT did not significantly improve clinical psychiatric symptoms and functioning. This indicates that the improvement in cognitive functioning from CRT is clear, but it is still necessary to translate outcome into “real-world” functional ability (9, 52). Considering that the dose of antipsychotics and anticholinergic burden may have an adverse effect on patients cognition (53, 54), we found no significant changes in CPZ-eq and ACB scores between baseline and follow-up. As the patient’s psychiatric symptoms may affect the change of cognitive function in CRT, our results demonstrates that the patient’s psychiatric symptoms remained relatively stabilized without additional antipsychotic medication adjustments during the CRT.

In the model of baseline demographic, clinical characteristics, neuropsychological test, and eye-movements factors, we found that schizophrenic patients with a baseline level of employment were more likely to benefit from intervention. Indeed, being employed is considered a capacity for premorbid adjustment and is associated with cognitive reserve (55). Previous findings have also confirmed that premorbid adjustment may be associated with the ability to recover from cognitive deficits through CRT, and CRT combined with employed status can reduce the negative impact of cognitive impairment on work performance (56, 57). This suggests that patients in employed status are at a more appropriate time for CRT.

In terms of eye-movement characteristics, in addition to the mean latency (overlap/gap/step) of prosaccade task, we also calculated the latency difference between the overlap and the gap condition (gap effect). In the overlap condition, attention remained at the initial point of origin when the target stimulus appeared. In the gap condition, attention was disengaged from the central point before the target stimulus appeared, making a faster response to the target. The reduction in latency during prosaccade under gap conditions compared with overlap conditions is described as the gap effect (58). Deficits in visual attention separation may be reflected by increased gap effects (59). Schizophrenia is associated with deficits in visual attention (60, 61), and our results suggest that patients with a better visual attention performance at baseline may have more cognitive improvement after CRT, as these patients may be more likely to focus on tasks during the training. With regard to the error rate in the antisaccade, better performance at baseline predicts higher probability of cognitive improvement after treatment. It involves the ability of inhibition, cognitive control, and working memory (28, 62). It also provides a more direct and simple assessment of frontal lobe function, especially the dorsolateral prefrontal cortex (DLPFC) deficits (28). Compared with other eye-movement measures, it is more reflective of high-level cognitive abilities. Similar to the previous results, having a greater basal frontal thickness at baseline has been recognized as a predictor of CRT efficacy (18). Visual scanning is a biomarker of schizophrenia, and is associated with cognitive function (63). There were no significant associations between free-viewing variable and CRT outcomes in our study. Performance during this task may not only be related to cognitive function, but it may also be influenced by psychiatric symptoms (64), which were not significantly different between the two groups of patients.

At present, the relationship between baseline cognition and cognitive improvement outcomes after CRT is still mixed (15–17). Compared to neuropsychological test, we found that eye movement measurements are a more sensitive predictor of cognitive improvement in predicting improvement after CRT in patients with schizophrenia. Traditional neuropsychological scales are less likely to assess motor-related cognitive functions, but saccades measures can assess inhibition, spatial memory, and motor executive cognitive functioning. This advantage is supported by the results of our correlation analysis between baseline saccade measures and cognition scores. Compared with cognitive tests, eye movements measures broaden the range of brain regions tested and more sensitive to recognition of cognitive impairment (37). Meanwhile, neuropsychological tests need to be administered by trained clinicians and results could be affected by subjective factors of the participants. In contrast, eye movement measures rely on the equipment to record eye position and use of software to analyze the data, allowing for a more objective assessment of cognitive functions (64). Considering the convenience and economic advantages of measuring eye movements, eye movements have better clinical applicability than cognitive tests for predicting the efficacy of CRT. In these three eye-movement tasks, the combination of pro- and anti-saccade tasks were more suitable as assessments for predicting CRT effects compared to the free-viewing task.

There has been a lack of studies investigating the correlation between baseline cognitive status and improvement in specific cognitive domains. Our study showed that lower baseline T-scores were associated with greater improvement in processing speed, attention, and working memory domains. This means that predictions of intervention outcomes in these several cognitive domains still need to take into account baseline cognitive abilities. In addition to the previously mentioned factors, we also found that ACB score changes, baseline CPZ-eq, and number of training sessions influenced the improvement in different cognitive domains. Our results showed that having more training sessions was associated with working memory and executive functioning improvement. Training intensity is an important predictor of treatment response and is the most consistent finding in previous studies (65, 66). However, there have been no studies or guidelines recommending the number of training sessions needed for treatment to be effective, and the number ranges from 24 to 100 sessions in some trials (66). We found the possibility of achieving working memory improvement by reducing antipsychotics at baseline. This is consistent with previous results, as D_2_ blockade may downregulate prefrontal cortex D_1_ receptors and reduce the improvement of working memory (67). Although some studies have demonstrated that baseline serum anticholinergic levels are negatively predictive of overall cognitive function improvement (68), our results suggest that reducing the anticholinergic burden caused by psychiatric medications during the intervention could have a positive effect on the speed of information processing and executive functioning improvement.

There are several limitations to this study, First, the small sample size limits the number of covariates included in the logistic regressions to investigate more potential predictors. Our results need to be validated in larger samples in the future. Second, we lacked other cognitive domains tests (verbal memory and language function) in our neuropsychological battery, which could limit the effects of CRT. Third, we only assessed cognitive function at the end of training. We can investigate the trajectory of cognitive function over longer periods of time after CRT in order to understand more deeply the predictors of long-term cognitive improvement outcomes in the future.

## 5 Conclusion

In conclusion, there is still a lack of personalized interventions to meet the therapeutic goals of a wider range of patients in a clinical setting. We identified predictors of CRT improvement that not only took into account previous clinical and disease characterizations, but also included eye movements that can be easily evaluated in a clinical setting. Compared to neuropsychological testing, the combination of pro- and anti-saccade tasks is the sensitive method in predicting the cognitive improvement of CRT in patients. Meanwhile, investigating the predictors of improvement in different cognitive domains also provides future opportunities of more individualized and effective interventions for deficits in specific cognitive domains.

## Data availability statement

The raw data supporting the conclusions of this article will be made available by the authors, without undue reservation.

## Ethics statement

The studies involving humans were approved by The Ethics Committee of the Peking University Sixth Hospital. The studies were conducted in accordance with the local legislation and institutional requirements. The participants provided their written informed consent to participate in this study.

## Author contributions

JZ: Conceptualization, Formal analysis, Investigation, Writing – original draft, Data curation. JL: Data curation, Formal analysis, Writing – original draft. LZ: Software, Writing – original draft. LX: Software, Writing – original draft. CP: Investigation, Writing – original draft. BH: Investigation, Writing – original draft. QZ: Data curation, Formal analysis, Writing – original draft. YL: Investigation, Writing – original draft. YT: Software, Writing – original draft. LY: Investigation, Writing – original draft. CS: Funding acquisition, Project administration, Writing – review & editing.
