# Supplementary material for: Eye movements as predictor of cognitive improvement after cognitive remediation therapy in patients with schizophrenia
Source: Front Psychiatry. 2024 Apr 16;15:1395198. doi: 10.3389/fpsyt.2024.1395198 (PMC11059054; doi:10.3389/fpsyt.2024.1395198)
Supplement: Supplementary file 1 [file DataSheet_1.docx]

**SUPPLEMENTARY MATERIALS**

**Eye movement as Predictors of Cognitive Improvement after Cognitive Remediation Therapy in Patients with Schizophrenia**

**Zhu JH, et al.**

**Table of contents** page

Supplementary Figure S1. User interface schematic of training games software applications........... 2

Supplementary Figure S2. The correspondence between game tasks and cognitive domains in the training system...................................................................................................................................... 2

**
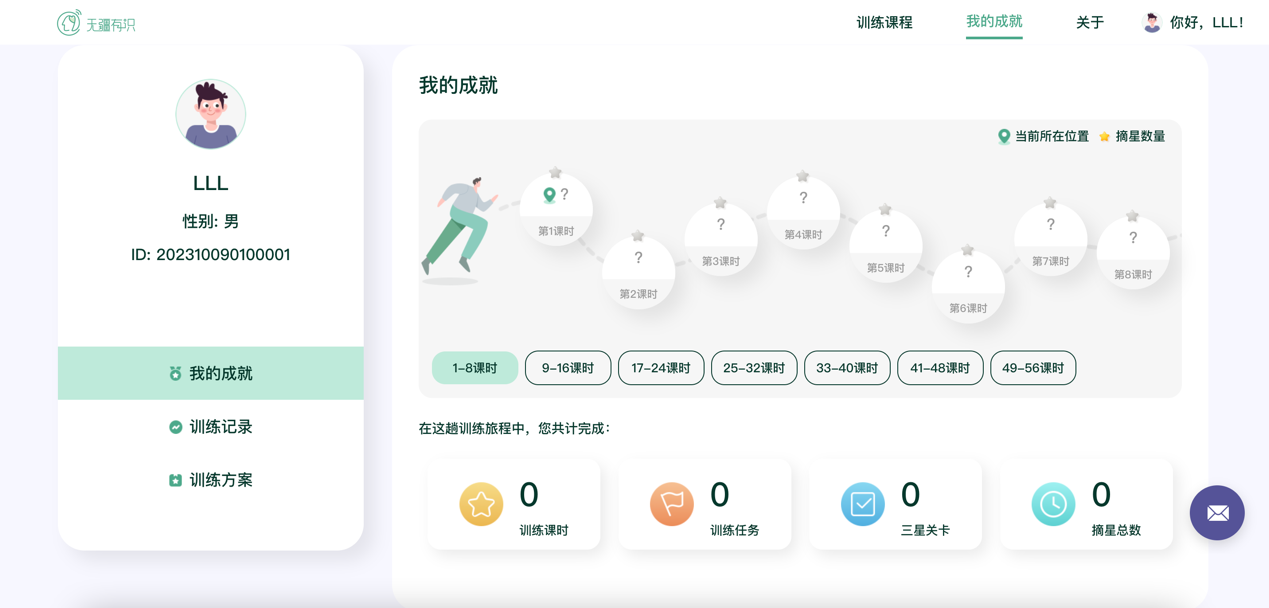
**

**Figure S1.** User interface schematic of training games software applications.


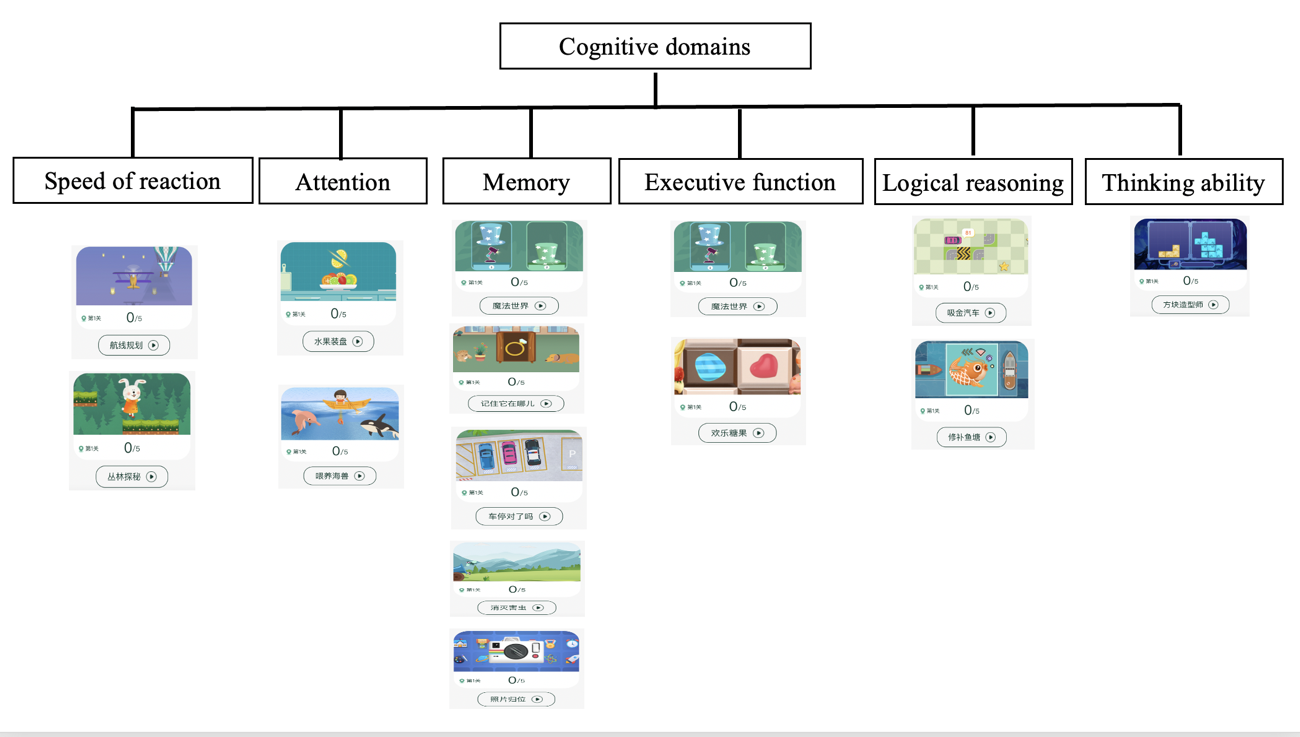


**Figure S2.** The correspondence between game tasks and cognitive domains in the training system.
